# Metabarcoding-based fungal diversity on coarse and fine particulate organic matter in a first-order stream in Nova Scotia, Canada

**DOI:** 10.12688/f1000research.7359.2

**Published:** 2016-02-26

**Authors:** Christian Wurzbacher, Ivan J. Grimmett, Felix Bärlocher

**Affiliations:** 1 Leibniz-Institute of Freshwater Ecology and Inland Fisheries (IGB), Berlin, Germany; 2 Department of Biological and Environmental Sciences, University of Gothenburg, Gothenburg, Sweden; 3 Department of Biology, Mt. Allison University, Sackville, NB, Canada

**Keywords:** aquatic fungi, stream, pyrosequencing, CPOM, FPOM

## Abstract

Most streams receive substantial inputs of allochthonous organic material in the form of leaves and twigs (CPOM **, **coarse particulate organic matter). Mechanical and biological processing converts this into fine particulate organic matter (FPOM). Other sources of particles include flocculated dissolved matter and soil particles. Fungi are known to play a role in the CPOM conversion process, but the taxonomic affiliations of these fungi remain poorly studied. The present study seeks to shed light on the composition of fungal communities on FPOM and CPOM as assessed in a natural stream in Nova Scotia, Canada. Maple leaves were exposed in a stream for four weeks and their fungal community evaluated through pyrosequencing. Over the same period, four FPOM size fractions were collected by filtration and assessed. Particles had much lower ergosterol contents than leaves, suggesting major differences in the extent of fungal colonization. Pyrosequencing documented a total of 821 fungal operational taxonomic units (OTU), of which 726 were exclusive to particles and 47 to leaf samples. Most fungal phyla were represented, including yeast lineages (e.g., Taphrinaceae and Saccharomycotina), Basidiomycota, Chytridiomycota and Cryptomycota, but several classes of Pezizomycontina (Ascomycota) dominated. Cluster dendrograms clearly separated fungal communities from leaves and from particles. Characterizing fungal communities may shed some light on the processing pathways of fine particles in streams and broadens our view of the phylogenetic composition of fungi in freshwater ecosystems.

## Introduction

Headwaters are almost entirely heterotrophic – up to 99% of their energy is supplied by coarse organic matter (CPOM, diameter > 1 mm) imported from the terrestrial surroundings (e.g., twigs, branches, and leaves). These allochthonous sources are converted into fine particulate organic matter (FPOM) mechanically by the water current, by feeding activities of invertebrate shredders (both by “sloppy” feeding and by feces production due to incomplete digestion; Cummins & Klug, 1979; Shepard & Minshall, 1981; Wotton *et al.*, 1998) and by fungal maceration ( Suberkropp & Klug, 1980). Stream fungi are vitally important for energy transformation of submerged leaf litter ( Baldy *et al.*, 1995; Gessner & Chauvet, 1994; Gulis & Suberkropp, 2003; Hieber & Gessner, 2002). FPOM may also be blown in or washed in from adjacent forest soils, or originate from sloughed-off algal biofilms, consist of plant spores and pollen ( Czeczuga & Orlowska, 2001), or be produced by flocculation of DOM, with or without microbial participation ( Lush & Hynes, 1973; Wotton, 1990). Due to the many biological processes involving CPOM and FPOM, bacteria and fungal spores will also contribute to the pool of stream FPOM ( Bärlocher & Brendelberger, 2004; Callisto & Graça, 2013; Edwards & Meyer, 1987; Gleason *et al.*, 2009). FPOM is one of the major components of stream ecosystems, and entire groups of organisms, such as the filter feeding guild, depend on it ( Callisto & Graça, 2013; Wallace & Merritt, 1980).

However, very little is known about FPOM associated microbial communities. Fine particles, regardless of their origin, are continually colonized and transformed by microorganisms. Due to resource limitation on small particles, we can assume that the biomass of mycelial fungi, as measured by ergosterol concentrations ( Callisto & Graça, 2013; Findlay *et al.*, 2002), will be low and that zoosporic and/or unicellular fungi will be more prominent due to their adaptations to small substrates such as algae and pollen ( Gleason *et al.*, 2008). Previous studies have shown that ascomycetous hyphomycetes are dominant stream dwelling fungi on leaf-litter (e.g. Bärlocher, 1990; Duarte *et al.*, 2015). Some of these leaf-litter fungi survive passage through the gut of leaf-eating amphipods ( Bärlocher, 1981; Sridhar *et al.*, 2011), and DNA from both ascomycetes and chytridiomycetes is present in fecal particles ( Sridhar *et al.*, 2011).

The present paper seeks to examine the fungal community on collected stream FPOM and whether it is possible to use it as a sum parameter for various fungal processes in the stream ecosystem. For this feasibility study we collected three FPOM size fractions and compared them with four weeks old submerged leaf-litter. We measured ergosterol as a biomass indicator of Dikarya and employed a metabarcoding approach in order to classify the fungal community of stream organic matter (OM).

## Methods

The field experiment was conducted in Boss Brook, a small stream in Fenwick, Nova Scotia, Canada (45° 43.00'N, 64° 09.56'W) ( Nikolcheva *et al.*, 2003). This first-order stream runs through a mixed forest dominated by white birch ( *Betula papyrifera* Marsh), several maple species ( *Acer rubrum* L., *Acer saccharum* Marsh., *Acer spicatum* Lam.), and white spruce ( *Picea glauca* [Moench] Voss). The stream bed consists of stones and gravel. At the sampling site, the stream is 2 to 3 m wide and 20 to 50 cm deep ( Grimmett *et al.*, 2012). On three dates (27 September, 25 October, 7 November 2011), 100 l of stream water were passed through a stack of metal filters, yielding 4 FPOM fractions (fraction 1: 2–1 mm; fraction 2: 1–0.5 mm; fraction 3: 0.5–0.25 mm, fraction 4: 0.25–0.020 mm). Most material was recovered in the lowest size fraction (4) and no material was recovered in fraction 1 ( Dataset 1). Samples were lyophilized and weighed. Additional samples for ergosterol measurements were collected on 12 November. These were freeze-dried and stored in methanol/potassium hydroxide (3 × 15 mg in 2 ml each at – 20°C; Nikolcheva *et al.*, 2003, Dataset 2). Samples of different dates were combined for pyrosequencing in order to adjust for the temporal variation and to increase the resolution, resulting in one sample each for size fractions 2 to 4. In parallel, senescent leaves from individual trees (Maple: *Acer platanoides*) were incubated as leaf discs (15 cm) in duplicate bags in the stream for four weeks (11 October to 9 November, 2011) to evaluate fungal communities on CPOM (procedures as in Grimmett *et al.*, 2012). As an (aquatic) outgroup we incubated leaves from a non-native tree (European beech, *Fagus sylvatica*) in the littoral zone of a lentic system (Lake Utopia, near St. George, NB, Canada; 45° 10.18'N 66° 47.67'W) for two weeks. The goal was to obtain an indication if substrate type (leaf vs. fine particles) may be more important than species (maple vs. beech) or habitat (lotic vs. lentic). All leaf samples were stored at -20°C until DNA extraction. In total we sequenced 6 samples consisting of two replicates (Maple I and Maple II) from the leaf bags, one lake-derived beech leaf bag sample, and one sample per stream-particle sample.

DNA from freeze-dried particle fractions and frozen leaf samples was extracted with the PowerSoil MoBio Kit as per the manufacturer's instructions (leaves were first cut into smaller pieces with a sterile scalpel). Amplicon PCR was performed using the barcoded 18S primers nu-SSU-0817 and nu-SSU-1536 of Borneman & Hartin (2000), and AccuPrime High Fidelity Polymerase (Life Technologies # 12337016) in a two-step PCR for 32 cycles (94°C for 1 min and 60°C for 4 min) with an initial denaturation for 5 min; BSA was added at a final concentration of 0.9 µg µl ^-1^. Amplicons were prepared according to the Lib-L protocol (454 Life Sciences, Roche) and sequenced by a benchtop GS Junior System (454 Life Sciences, Roche). Raw data were processed by Mothur (version 1.26, Schloss *et al.*, 2011), following recommendations for standard operating procedure ( http://www.mothur.org/wiki/Schloss_SOP, accessed 7/2012), implementing denoising, trimming, alignment, filtering, chimera removal, classification, and preclustering steps against the eukaryotic reference database provided by Mothur. OTUs were calculated on a 97% basis of a final alignment with a median length of 279 nt and statistics (diversity estimates and similarities) were done with a random submatrix normalized to the lowest number of reads. After the filtering process and removal of chimeric sequences, there were 10,000–22,000 reads per sample, resulting in a sampling coverage of > 99%. The OTU table ( Dataset 3) for those reads was exported to R (version 2.15.1) for cluster analysis with the second Kulczynski similarity index ( http://cran.r-project.org/). In addition, representative OTUs of the associated FASTA file were realigned with SINA (version 1.2.11, Pruesse *et al.*, 2012) and imported into the SILVA SSU reference database version 111 ( http://www.arb-silva.de/). The representative sequences from all OTUs were added to the SILVA reference database by the parsimony option activating the eukaryotic positional variability filter implemented in ARB (version 5.5, Ludwig *et al.*, 2004). The resulting tree (i.e. the top 100 subtree) was exported and processed by FigTree (version 1.4, http://tree.bio.ed.ac.uk/software/figtree/). The raw sequences were deposited in the European Nucleotide Archive (ENA; accession number PRJEB10809).

## Results

Amount of particles in the stream Dataset 1 provides the amounts of particles (mg) per liter of stream water. The particle size is defined as written in the method section for F2–F4. Click here for additional data file. Copyright: © 2016 Wurzbacher C et al. 2016 Data associated with the article are available under the terms of the Creative Commons Zero "No rights reserved" data waiver (CC0 1.0 Public domain dedication).

Ergosterol content of particle size fractions Dataset 2 provide the ergosterol content (µg) per mass of particles or leaf-species (g) as described in the method section. Click here for additional data file. Copyright: © 2016 Wurzbacher C et al. 2016 Data associated with the article are available under the terms of the Creative Commons Zero "No rights reserved" data waiver (CC0 1.0 Public domain dedication).

OTU matrix including fasta sequences Dataset 3 is the resulting OTU matrix after the sequencing data processing with Mothur as described in the method section. For each OTU the amount of reads per individual sample is given. In addition one representative read is given (Representative_read_id) and the corresponding aligned DNA sequence. Click here for additional data file. Copyright: © 2016 Wurzbacher C et al. 2016 Data associated with the article are available under the terms of the Creative Commons Zero "No rights reserved" data waiver (CC0 1.0 Public domain dedication).

Average stream FPOM concentration of the three sampling dates was 2.7 mg l ^-1^. Distribution among the various size fractions is summarized in Table 1. Due to fluctuating water flow, there was a high temporal variation in the amount of recovered particles. On average the smallest size fraction (250 µm – 20 µm) was the most abundant. Ergosterol concentrations decreased with lower particle size ( Table 1). It was highest on maple leaves recovered from Boss Brook and also higher on our lentic outgroup: beech leaves from Lake Utopia.

**Table 1.** Mass of FPOM fractions (mg l ^-1^, mean ± SD; n = 3) and ergosterol concentrations (µg g ^-1^, mean ± SD) of FPOM and beech (Utopia Lake) and maple (Boss Brook leaves). Ergosterol values were evaluated by ANOVA (p < 0.0001), followed by Tukey-Kramer. Averages with same letter are not significantly different (p > 0.05). Based on Dataset 1 and Dataset 2.

FPOM	Fraction (mm)	Mass (mg l ^-1^)	Ergosterol (µg g ^-1^)
	F4 (0.25-0.02)	2.7±2.5	0.03 ^a^ ± 0.01
	F3 (0.5-0.25)	0.01±0.02	0.52 ^a,b^ ± 0.07
	F2 (1-0.5)	0.01±0.02	1.2 ^b^ ± 0.2
CPOM	Beech	na	2.9 ^c^ ± 0.7
	Maple	na	35.5 ^d^ ± 1.1

**Figure 1.**
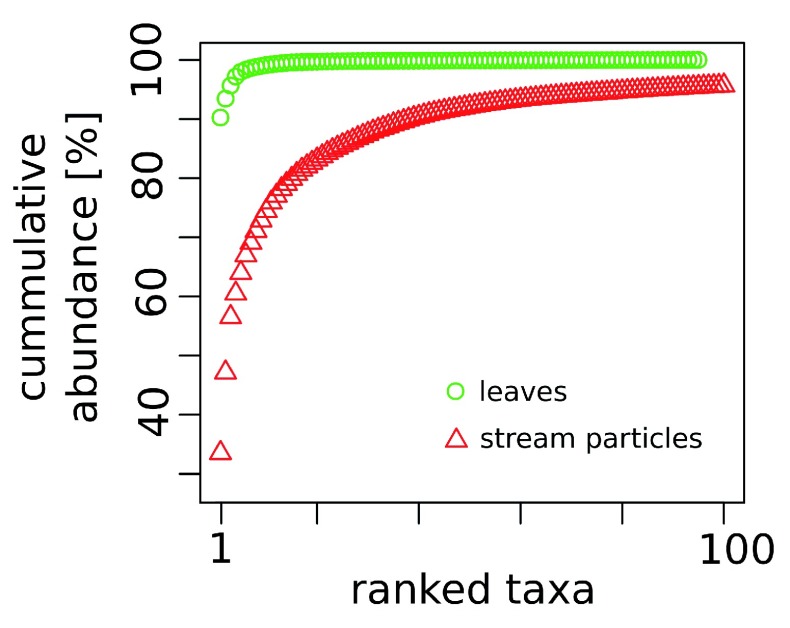
Rank abundance curves for the dominant 100 OTUs of leaves and stream particles, all leaf samples (beech and maple samples) and all size fractions were combined into the two categories “leaves” and “stream particles” (based on Dataset 3).

**Figure 2.**
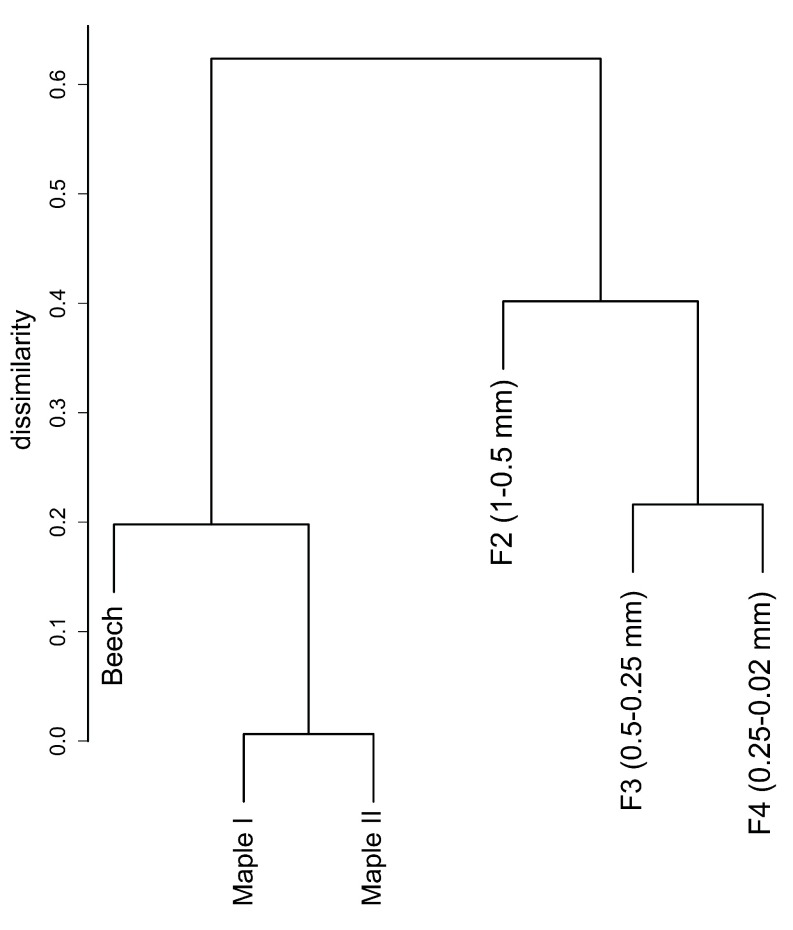
Cluster dendrogram of the fungal community composition data presenting the dissimilarity of the leave samples and the particle size fractions based on pyrosequencing. Data based on a Kulczynski similarity matrix, clustered with UPGMA (average) method (based on Dataset 3).

**Figure 3.**
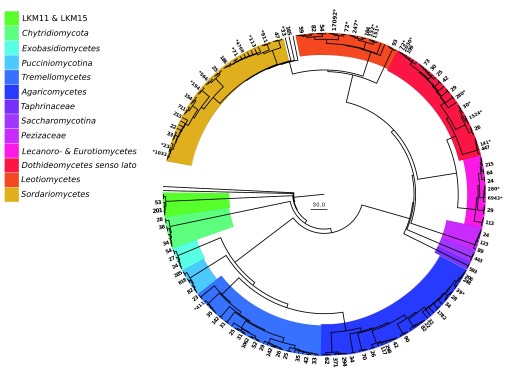
Phylogenetic tree based on the SILVA reference database and the top 100 OTUs retrieved from particle DNA (added with parsimony). Numbers of retrieved sequences per OTU are written at the outer rim; asterisks mark taxa that were also recovered on leaf litter. Branches without numbers are reference sequences (SILVA) (based on Dataset 3).

The DNA sequences were assigned to 821 fungal OTUs. Of these, 726 were detected exclusively in the particle fractions, and 47 (out of 95 OTUs detected on leaves) were restricted to leaf samples. The particles shared 130 OTUs (with an extrapolated shared species Chao index of 215.5). The taxon richness of the particles was an order of magnitude higher than the one of maple leaf samples and the inverse Simpson index pointed to a more even and diverse community structure on particles than on leaves ( Table 2). This is further reflected in the rank abundance curves of the two substrate types ( Figure 1). Correspondingly, the analysis of fungal communities separates leaves from particles with less than 40% similarity ( Figure 2). When we looked at the most prominent 100 OTUs from stream particles, which accounted for 96% of all sequences ( Figure 1), we found representatives of most fungal phyla including yeast lineages (e.g. Taphrinaceae and Saccharomycotina), Basidiomycota, and the phyla Chytridiomycota and Cryptomycota ( Figure 3). The fungal diversity was dominated by several classes of Pezizomycotina (Ascomycota).

**Table 2.** Number of sequences (nseqs), coverage (cov), number of observed taxonomic units (otu), inverse Simpson index (invsim), and estimated richness as Chao index (chao) for leaf and particle fractions F2 to F4 (based on Dataset 3).

Substrate	nseqs	cov	otu	invsim	chao
F2 (1 – 0.5 mm)	18343	0.992	356	7.719	445.8
F3 (0.5 – 0.25 mm)	16375	0.990	429	6.890	523.3
F4 (0.25 – 0.02 mm)	16248	0.988	456	4.649	613.5
Maple I	9884	0.999	20	1.025	34.0
Maple II	17444	0.999	27	1.025	28.3
Beech	22267	0.998	72	1.549	115.0

In order to trace back fungal taxa derived from leaf-litter decomposition (marked with asterisks in Figure 3), we looked at the most abundant OTUs on the maple leaves incubated in the stream ( Table 3). OTU 1 (Pezizomycotina) was the most abundant OTU in all samples. On average between 62.0 – 72.1% of the taxa found on each of the FPOM fractions were also present on submerged leaf litter. To get rough estimates of alternative origins and functions of the fungal communities on stream particles, we split them into different categories: potential soil fungi (with Agaricomycetes as proxy) accounted for 4.8 – 12.3%, yeast-like fungi made up 5.3 – 6.8%, and non-Dikarya fungal lineages ranged between 0.8 – 1.6%.

**Table 3.** Comparison of OTUs on maple leaves (mean of replicates), FPOM (mean of size fractions) and beech leaves with standard deviation when applicable. The table is sorted after the most abundant OTUs on maple leaves in descending order. (n.d. = not detected, based on Dataset 3).

OTU	Maple (%)	FPOM (%)	Beech (%)
1	98.79 ± 0.01	33.82 ± 7.39	79.79
75	0.35 ± 0.11	3.98 ± 0.41	n.d.
129	0.19 ± 0.08	0.14 ± 0.04	n.d.
4	0.15 ± 0.15	1.11 ± 0.25	4.77
3	0.13 ± 0.08	8.9 ± 9.90	6.98
11	0.11 ± 0.10	13.94 ± 6.95	0.18
96	0.04 ± 0.02	0.3 ± 0.29	n.d.
23	0.04 ± 0.05	0.12 ± 0.09	0.02
338	0.04 ± 0.01	0.01 ± 0.01	n.d.
6	0.02 ± 0.01	0.13 ± 0.17	0.82

## Discussion

In this study we focussed on stream particles and we were especially interested in the fungal phyla we may find on them. Thus we applied a conservative marker gene, which is especially efficient at resolving the basal branches of Fungi ( Mohamed & Martiny, 2011). With this we could successfully detect a broad spectrum of fungal phyla on FPOM, including Chytridiomycota sequences and taxa belonging to the newly described group of Cryptomycota ( Jones *et al.*, 2011). Chytridiomycota have been documented on leaf litter in freshwater streams before ( Bärlocher *et al.*, 2012; Marano *et al.*, 2011; Nikolcheva & Bärlocher, 2004), but, to our knowledge, this is the first study to document Cryptomycota in streams ( Jones *et al.*, 2011). Cryptomycota are assumed to be parasitic on various organisms including fungi ( Gleason *et al.*, 2012), however, some evidence also points to a saprobic life style ( Wurzbacher *et al.*, 2014). Their ecological role in streams needs to be further elucidated, especially since they have the potential for mycoparasitism. Some aspects of their occurrence and ecology have been summarized by Grossart *et al.* (2016). We did not find typical trichomycete sequences (e.g. Lichtwardt, 1972), which would have pointed to a gut passage of particles through filter-feeders. Possibly, the sampling sites had an insufficient number of filter feeders or too few gut fungi on fecal pellets to allow detection using our methods.

The high diversity of fungi on stream particles stands in contrast to the very low diversity on leaf-litter. It is likely that the dominant OTU 1 comprises several prominent aquatic hyphomycete species, since these rarely differ in their nuclear SSU sequence ( Belliveau & Bärlocher, 2005, see also Tedersoo *et al.*, 2015 for general limitations of SSU for Dikarya). In general we think that the high diversity on particles reflects their multiple origins and histories. The fact that those few leaf-litter taxa were also abundant in the stream particles points to leaf-litter as one important particle origin. For example, if the stream is dominated by particles washed in from the forest we would have expected Basidiomycota to dominate ( Tedersoo *et al.*, 2014). They accounted for 72% in Lim *et al.* (2010) and for ≥ 60% in Shi *et al.* (2014) with Agaricomycetes as the most common class, a much larger proportion than on leaves or particles in the current study (4.8 – 12.3%). However, it is also conceivable that soil particles, upon immersion in a stream, undergo further processing during which Basidiomycota are rapidly replaced by indigenous stream fungi. The transport and age of FPOM and its distribution among various size classes is highly variable and strongly depends on hydrological fluctuations throughout the seasons ( Bilby & Likens, 1979; Thomas *et al.*, 2001). In other words, it may not possible to deduce the origin of the stream FPOM by looking at taxonomic composition of its mycoflora. But the high fungal diversity on stream particles points to the interaction of various stream processes. In this context it is interesting to compare our findings with an arctic study which focussed on unfractionated water samples ( Crump *et al.*, 2012) that showed that only a minor fraction of eukaryotic microorganisms (< 10%) in a first order stream originated from the soil, while the majority seemed to be indigenous. Interestingly, the situation was the reverse for prokaryotes, which were predominantly washed in from soil ( Crump *et al.*, 2012).

 In our study, ergosterol concentration decreased in smaller particles, pointing to reduced biomass of living fungi derived from e.g. leaf-litter. Still, fungal OTUs found on submerged CPOM (leaf-litter) dominate the sequences on all particle size classes. This suggests that most fungal taxa were reduced non-selectively during the processing of CPOM to FPOM, or that the fungal cells were degraded (as suggested by the decline of ergosterol) but their DNA remained largely intact and attached to the particles as environmental DNA. Such bound DNA can remain stable for extended periods of time ( Guggenberger & Kaiser, 2003; Nguyen & Elimelech, 2007) and it is known that extracellular DNA occurs in considerable quantities in aquatic systems and sediments (summarized in Pietramellara *et al.*, 2009).

In order to test the hypothesis that the fungal community of FPOM is indeed a function of present stream processes, the storage potential of FPOM has to be defined accurately by investigating the fungal taxa turnover on particles of known origin and composition incubated in a stream. Supplementing these approaches with ribosomal RNA will allow us to control for the proportion of environmental DNA. Much of the biological processing of FPOM in streams is still unclear ( Tank *et al.*, 2010), and tracking their DNA levels and diversity might shed some light on their origin, history and in-stream transport.

## Conclusions

We successfully looked at the broad phylogenetic diversity of stream FPOM (> 20 µm), which was much higher than on leaf-litter and included members of novel groups (Cryptomycota). The most abundant operational taxonomic units on particles were identical to taxa on submerged decomposing leaf litter. We documented distinct differences in ergosterol content between particle sizes, which points to the near absence of living Dikarya mycelium on smaller stream particles. Both fungal diversity and community composition differed significantly between the two substrate types. Nevertheless, more extensive efforts will be required to unravel the relative effects of origin (e.g., leaves decomposing in the stream vs. soil particles vs. algal particles) and processing or ageing of particles including DNA storage within the fungal community. Combining this information should allow us to more fully document various stream processes initiated by fungal organism.

## Data availability

The data referenced by this article are under copyright with the following copyright statement: Copyright: © 2016 Wurzbacher C et al.

Data associated with the article are available under the terms of the Creative Commons Zero "No rights reserved" data waiver (CC0 1.0 Public domain dedication). 

Raw sequence data for the samples reported here can be found in the European Nucleotide Archive ( http://www.ebi.ac.uk/ena), under accession PRJEB10809.

F1000Research: Dataset 1. Amount of particles in the stream, 10.5256/f1000research.7359.d107489 ( Wurzbacher *et al.*, 2015a).

F1000Research: Dataset 2. Ergosterol content of particle size fractions, 10.5256/f1000research.7359.d107490 ( Wurzbacher *et al.*, 2015b).

F1000Research: Dataset 3. OTU matrix including fasta sequences, 10.5256/f1000research.7359.d107491 ( Wurzbacher *et al.*, 2015c).
