# Supplementary material for: Metabarcoding-based fungal diversity on coarse and fine particulate organic matter in a first-order stream in Nova Scotia, Canada
Source: F1000Res. 2016 Feb 26;4:1378. Originally published 2015 Nov 30. [Version 2] doi: 10.12688/f1000research.7359.2 (PMC4755416; doi:10.12688/f1000research.7359.2)
Supplement: Peer review history

# Peer review: Referee response for version 1

**DOI:** 10.5256/f1000research.7930.r12451

**Published:** 2016-02-15

**Authors:** Huzefa A Raja

**Affiliations:** 1 Department of Chemistry and Biochemistry, University of North Carolina, Greensboro, NC, USA

This is a very well written manuscript. I do not have much to comment, except that this is a well thought out experimental study. It is very interesting to note that a larger number of OTUs were obtained from FPOM, whereas, the OTUs from CPOM (leaf litter) were fewer in number. Finding members of Cryptomycota in streams for the first time was interesting. I would have liked to see the ITS region sequenced, so we could get a better understanding of the species-level identification.

I have read this submission. I believe that I have an appropriate level of expertise to confirm that it is of an acceptable scientific standard.

# Peer review: Referee response for version 1

**DOI:** 10.5256/f1000research.7930.r12182

**Published:** 2016-02-03

**Authors:** Belle Damadora Shenoy

**Affiliations:** 1 CSIR-National Institute of Oceanography Regional Centre, Visakhapatnam, India

The manuscript is well-written, and will be acceptable after a few minor suggestions are incorporated:

 

- The abstract should contain more information on the results of the paper.
- I would rather like to see (in the abstract) how much light your pyrosequencing has shed on the origins and processing pathways of fine particles.
- In the discussion section, the authors write  “[…] to our knowledge, this is the first study to document Cryptomycota in streams (Jones *et al.,* 2011).” – the authors could highlight recent reports on Cryptomycota from fresh water bodies.
- In the conclusions, the authors write “In other words, it is not possible to deduce the origin of the stream FPOM by looking at taxonomic composition of its mycoflora.”. This is a possible contradiction with the last sentences of the abstract and discussion sections?

These suggestions are also available as an annotated PDF.

I have read this submission. I believe that I have an appropriate level of expertise to confirm that it is of an acceptable scientific standard.

# Response

**Published:** 2016-02-22

**Authors:** Christian Wurzbacher

**Affiliations:** University of Gothenburg, Sweden

Thank you very much for your review. We amended your earlier suggestions in Version 2 of the article:

"In this version we followed the suggestions of the one of the referee reports by (i) adding more results to the abstract, (ii) giving another reference for recent discussions on Cryptomycota in freshwater systems, and by (iii) consistently stating that it may not be possible to deduce the origin of the particle by analysing its fungal community."

With kind regards
